# A Reliable Low-Cost Three-Dimensional (3D)-Printed Brachial Plexus Model for Enhanced Anatomical Learning

**DOI:** 10.7759/cureus.104662

**Published:** 2026-03-04

**Authors:** Manuel Cevallos, Joshua J Meyer, Jackson Beeching, Jad Mazboudi

**Affiliations:** 1 Surgery, University of Arizona College of Medicine - Phoenix, Phoenix, USA; 2 Medical Education, Creighton University School of Medicine, Phoenix, USA

**Keywords:** 3d-printed model, 3d-scanning, anatomy, brachial plexus, medical education, spatial visualization

## Abstract

The brachial plexus is a complex network of nerve branches with a three-dimensional (3D) distribution, which can make it difficult for students at any level to identify its structures. Spatial visualization skills are required, but two-dimensional images are not ideal to learn about this neurological network. Despite the availability of commercial models and digital tools, substantial upfront costs, including but not limited to annual subscription fees, can pose a challenge for most resource-limited institutions, thus hindering effective learning. This technical report proposes a practical and cost-effective solution.

Researchers demonstrate how direct 3D scanning of cadaveric structures is used to create an affordable 3D-printed model that captures key anatomical details. The 3D brachial plexus model preserves the traditional structure (no variants), facilitating student learning and enabling use in the lab or at home. In a brief pilot study using a voluntary, anonymous survey with Likert-style questions (institutional review board (IRB)-approved), the model was tested with current physician assistant students who took the anatomy course; their feedback is discussed in the article.

This model can enhance learning and help bridge the educational gap at low-resource universities. Researchers believe in the universality of education; for that reason, the 3D-printed brachial plexus model is available to any academic faculty interested in using and evaluating it in their anatomical courses.

## Introduction

The brachial plexus is a complex network of nerves that provides motor and sensory innervation to the upper limb. Knowledge of the brachial plexus helps understand the upper-limb innervation pattern, diagnose injuries (e.g., delivery vs. trauma), prevent surgical complications in the area, provide anesthetic blocks, and support rehabilitation for physical therapists and neurologists.

The brachial plexus has a complex structure that can be confusing for students because of its unusual spatial arrangement [1]. It features many branching patterns: roots, trunks, divisions, cords, and branches that cross and join to form new ones. That three-dimensional (3D) configuration, related to the main artery (axillary) and its branches and veins, poses a challenge for students at different educational levels in spatial visualization. Although commercial models are highly detailed, they often cost over $1000, making them impractical for schools to purchase in large numbers for entire classes and for students to use at home for self-learning [2]. Open-source digital models are available, but they may lack complete anatomical detail, limiting their educational utility. Lastly, the software requires a subscription, which limits students' access if their academic institutions do not cover it.

To address these issues, we developed a fast, scalable, and affordable process for creating high-quality 3D-printed models of the brachial plexus by directly scanning cadaveric anatomy at high resolution. 3D-printed anatomical models (3DPAMs) have been shown to improve both visual and tactile learning and increase student satisfaction [3].

The brachial plexus model was presented at the Tech Fair: Hands-on at the AACA 2025 Annual Meeting, held on June 16-19 in Bellevue, WA, USA.

## Technical report

Designing the 3D-printed model

**Figure 1:**
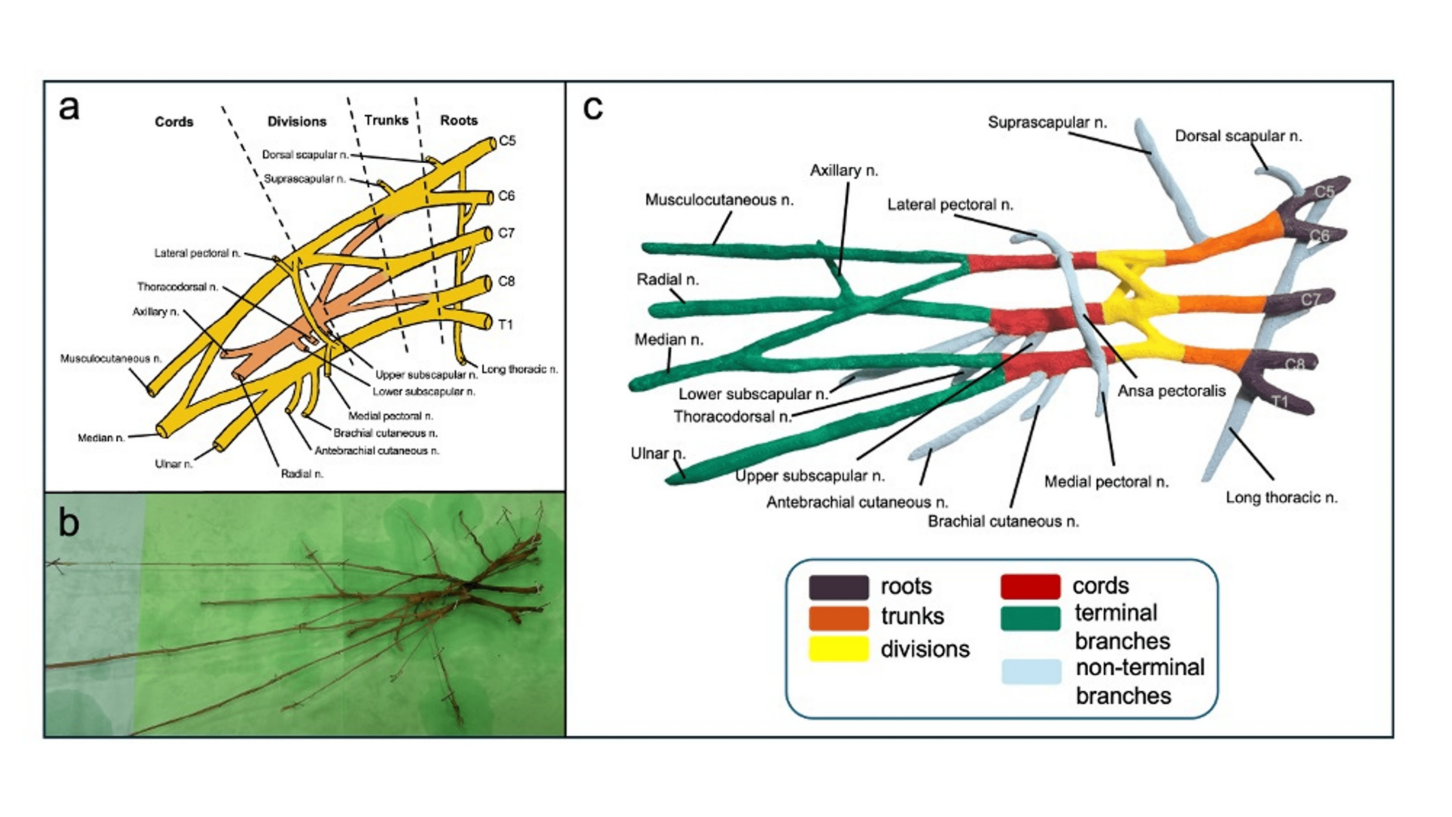
(a) Traditional diagram of the brachial plexus anatomy; (b) Isolated right brachial plexus resected from a cadaver; (c) Newly developed 3D-printed model of the brachial plexus with color-coded segments and labeled branches.

A cadaver, preserved in a 4% formaldehyde solution, from Creighton University (CU) School of Medicine’s Anatomy Lab in Phoenix, was selected after examining multiple specimens for optimal preservation and the absence of significant variation. The selected specimen displayed the typical pattern of root, trunk, division, cord, and branch arrangement, with most key branches intact. Initial attempts to scan the plexus in its natural position failed to provide enough detail; therefore, the plexus was carefully isolated and pinned to a Styrofoam block, maintaining its 3D shape and the relationships between its branches (Figure 1b).

A Revopoint MIRACO (Revopoint 3D Technologies Inc., Shenzhen, China), a quad-camera infrared structured-light 3D scanner, was used to generate a detailed 3D point cloud of the excised plexus. RevoScan-5 software (Revopoint 3D Technologies Inc., Shenzhen, China) was used to fuse and mesh the point cloud, resulting in a digital model that accurately captures the spatial relationships of the primary nerves. Some small branches, such as the upper subscapular nerve, were not captured by the scanner, and a few, such as the medial pectoral nerve, were not preserved in the dissection. Missing branches were digitally reconstructed and added to the model using Blender software (version 3.2.1; Blender Foundation, Amsterdam, The Netherlands), guided by anatomical references. The final model included all standard branches of the brachial plexus, as shown in Figure 1a.

The 3D model (Figure 1c) was printed using polylactic acid (PLA) filament on a Bambu Lab A1 printer (Bambu Lab, Shenzhen, China). The printed model was highly durable, accurately depicted standard anatomy, and cost less than $3 per unit to produce. The model, originally printed in a single color, was painted with acrylics to highlight the five segments and branches, enhancing its educational value.

The 3D-printed model was painted with the traditional color scheme used in undergraduate and healthcare textbooks; it primarily supports spatial recognition and, secondarily, the memorization of root contributions to branches. If the goal is to focus in detail on root contributions to branches, a better painting would use lines, similar to a “diagram of the subway train tracks”: one color for each root, with other roots included to reach each branch. Instructors can customize based on students' needs and the curriculum (Video 1).

**Video 1:** 3D-printed model of the brachial plexus.

Preliminary evaluation of the 3D-printed model

To evaluate the 3D-printed model, researchers conducted a pilot study using an anonymous, voluntary survey administered to first-year physician assistant students at CU who enrolled in the anatomy course in Fall 2025. The survey was previously approved by the CU Institutional Review Board (2005487). One model was distributed at each of 10 anatomical stations, and one student at each table volunteered; a total of seven students participated, answering Likert-style questions (1 = lowest/poor to 5 = highest/excellent) to evaluate the model's characteristics and the learning experience. Regarding the model's characteristics, all agreed that the size (20 cm), material (PLA), and wear/damage resistance were correct. For learning resources to visualize the brachial plexus, the most effective option was a cadaveric specimen; second was a 3D-printed model; third was a digital/virtual option; and last was images from the book (2D pictures). On a scale of one (poorest) to five (highest), using Likert-style questions, the data showed: the average for increasing knowledge retention was 4 of 5 (80%), increasing student confidence was 4.29 of 5 (86%), improving spatial visualization was 4.14 of 5 (83%), and helping prepare for a lab practical was 72%. The average of their answers was reported, and the main goal was to evaluate the model and acceptance for students in general. The next step in the project will be an assessment.

## Discussion

Using 3DPAMs offers several educational and practical advantages, including the production of realistic models at a low cost and support for effective anatomy teaching [4]. By capturing the actual spatial arrangement and preserving the proportions of the structures in the brachial plexus, the model enables students to grasp complex relationships in a tactile, 3D manner. Data from the pilot study support the model’s characteristics and show high student satisfaction, with knowledge retention, student confidence, and spatial visualization all exceeding 80%. It is not surprising that the model has a score below 80% for preparation for the lab practical, since that type of exam includes multiple variants and many of them are intrinsic to the student and to the complexity of questions across multiple organs simultaneously. To that end, we will need to re-evaluate the model solely with respect to the 3D brachial plexus model discussed. The data shown is the average percentage from the satisfaction survey, based on a limited sample size of 10 participants. The survey evaluated the model for students and collected feedback to move to the next step: assessing whether the model produces any change in knowledge. Another limitation of the model is the absence of the axillary artery; to address that, we built an axillary artery using pipe cleaners. It helps visualize the structure in that area, but it could be improved by building it with silicone resin (malleable and removable).

Unlike models created from imaging techniques such as CT or MRI, this approach bypasses time-consuming segmentation and post-processing steps, thereby reducing the time from dissection to a finished model. The scanning process is adaptable and can be used to create custom models that display anatomical differences observed during dissection. This is important because more than half of people have at least one variant branch in the plexus [5].

The main costs involve a one-time purchase of a 3D scanner and printer, while ongoing model production remains very affordable. This makes the process accessible even for institutions with limited budgets and encourages quick sharing of models between institutions. The 3D-printed brachial plexus model is available to anyone interested in using it in their anatomical classroom or evaluating it for research purposes (Interactive Model 1).

**Video 1:** 3D files of the brachial plexus model.

## Conclusions

Direct 3D scanning of cadaveric anatomy offers a practical, scalable approach for producing highly accurate, durable, and affordable 3DPAMs for anatomical education. By significantly reducing the costs and technical barriers associated with traditional model production, this approach enables institutions with limited resources to access high-quality teaching tools.

Ultimately, this innovation has the potential to democratize access to anatomical models, boost student engagement, and enhance understanding of complex structures, such as the brachial plexus, across diverse learning environments.
